# Effectiveness and safety of ultrasound-guided stellate ganglion block in C57BL/6 mice

**DOI:** 10.3389/fmed.2025.1679559

**Published:** 2025-12-11

**Authors:** Xiaohong Liu, Xuyang Wu, Liang Wu, Fengyi Lin, Jingjia Yan, Jiuyun Zhang, Xiaochun Zheng, Xiaohui Chen, Qingwang Lu

**Affiliations:** 1 Department of Anesthesiology, Shanghai Sixth People’s Hospital Fujian, Quanzhou, Fujian, China; 2 Department of Anesthesiology, Shengli Clinical Medical College of Fujian Medical University, Fujian Provincial Hospital, Fuzhou University Affiliated Provincial Hospital, Fuzhou, Fujian, China; 3 Department of Anesthesiology, Shanghai Yangpu District Central Hospital, Yangpu Hospital Affiliated to Tongji University, Shanghai, China; 4 Department of Anesthesiology, Fuzhou Second General Hospital, Fuzhou, Fujian, China; 5 Department of Emergency, Shengli Clinical Medical College of Fujian Medical University, Fujian Provincial Hospital, Fuzhou University Affiliated Provincial Hospital, Fuzhou, Fujian, China

**Keywords:** stellate ganglion block, nerve block, mice models, Horner’s syndrome, Ultrasound

## Abstract

**Objectives:**

Stellate ganglion block (SGB) is a unique anesthetic procedure distinguished by rapid onset, precise effects, and repeatability. The advent of ultrasound guidance has markedly increased the accuracy and safety of this technique, providing a novel approach for nerve blockade in model animals. This study aimed to evaluate the efficacy and safety of ultrasound-guided SGB in C57BL/6 mice, a strain commonly used in biomedical research because of its stable genetic characteristics and well-documented immune responses.

**Methods:**

A total of 8–10-week-old C57BL/6 mice were used in this study and were divided into three groups: the control group, the SGB-R group (right side), and the SGB-L group (left side). The SGB-R and SGB-L groups received an injection of 0.25% ropivacaine solution in a volume of 0.08 mL, whereas the control group was administered an equivalent volume of saline. To evaluate the efficacy of the procedure, we monitored the incidence of Horner’s syndrome, heart rate fluctuations, changes in carotid artery flow velocity and diameter, and temperature variations in the affected upper limb. Additionally, we used 3D CT imaging to precisely identify the needle tip position and the diffusion range of the local anesthetic. Simultaneously, we documented the associated complications, including brachial plexus block, hematoma, respiratory distress and mortality, to assess the safety of the procedure.

**Results:**

Among the SGB-treated mice, 100% presented with Horner’s syndrome. Compared with preintervention levels, the SGB-R and SGB-L groups presented significant decreases in heart rate, increases in carotid artery diameter, increased blood flow velocity, and elevated limb temperature on the blocked side after SGB intervention. Compared with the Con group, the SGB-R and SGB-L groups presented significantly greater carotid artery diameter and blood flow velocity, as well as notable increases in limb temperature. Importantly, no major postsurgical complications, such as brachial plexus injury, hematoma, respiratory distress, or mortality, occurred in any of the groups.

**Conclusion:**

This study presents a methodological blueprint for the implementation of ultrasound-guided SGB in C57BL/6 mice, demonstrating its potential effectiveness and safety. The newly established SGB model significantly enhances stability and minimizes potential complications. Compared with traditional techniques, this method offers superior applicability for SGB-related research.

## Highlights

- Ultrasound-guided SGB is successfully established with a low complication rate in a mouse model.
- Establishes a safe and effective model for studying the mechanisms and systemic effects of SGB. The ultrasound device is positioned at the level of the first rib to visualize and identify the carotid artery, trachea, and structure of the first rib. The needle is inserted below the carotid artery and adjacent to the trachea. After ensuring there is no blood return upon aspiration, the local anesthetic is injected.

## 1 Introduction

The stellate ganglion (SG) is a sympathetic ganglion formed by the fusion of the inferior cervical ganglion and the first thoracic ganglion. It is located at the base of the transverse process of the seventh cervical vertebra (C7) and on the front of the neck of the first rib. The SG contains sympathetic preganglionic fibers supplying the head and neck, as well as postganglionic fibers that supply the upper limb and the heart (1, 2). The preganglionic neurons of the stellate ganglion are located in the lateral horns of the spinal cord from T1 to T2. The preganglionic neurons exit the central nervous system through the anterior roots of the spinal nerves and the white rami communicantes. They synapse in the SG, subsequently giving rise to postganglionic fibers that connect with the spinal nerves and form anastomoses with each other. The SGB procedure involves injecting a local anesthetic near the SG. SGB is indicated for conditions such as hot flashes, sleep disorders (3), arrhythmia (4), complex regional pain syndrome (5), and posttraumatic stress disorder (6).

Given the widespread clinical application of SGB, it is crucial to understand its intrinsic mechanisms, and establishing an effective and safe animal model is essential for further research. Rats are most commonly used because their SG is anatomically similar to that of humans (7, 8). Compared to rat models, mice models offer several distinct advantages, including a wider array of transgenic, gene knockout, and gene knock-in options, as well as a greater variety of strains for developing experimental models. Consequently, establishing an SGB model in mice is highly important. However, mice have a small neck structure, which presents challenges in accurately identifying neuroanatomical locations and increases the risk of injury to surrounding tissues. Ultrasound enables clear visualization of the anatomical structures while concurrently allowing the monitoring of needles, catheters, and drug diffusion. This significantly improves success rates and reduces the rates of complications associated with target nerve block (7). Ultrasound-guided SGB has undergone rigorous validation through numerous clinical trials in human subjects (9, 10), consistently demonstrating a significant reduction in adverse reactions and in complications such as intravascular injections, laryngeal nerve paralysis and injury (11). Therefore, the aim of this study was to develop a detailed ultrasound-guided SGB technique in C57BL/6 mice and to systematically evaluate its effectiveness and safety, thereby establishing a standardized and precise animal model.

## 2 Materials and methods

### 2.1 Research approval and mice

The research received approval from the Animal Experimentation Committee of Fujian Provincial Hospital (No. IACUC-FPH-SL-20240813[0343]). The resource equation method is an optimal approach for estimating the sample size in exploratory animal studies. The formula used is E = N−K = K⋅ n−K = K (n−1), where 10 ≤ E ≤ 20, *N* is the total sample size, *n* is the sample size per group, *K* is the number of treatment groups, and *E* represents the error degrees of freedom. This can be simplified to *n* = (E/K) + 1. Consequently, the required sample sizes are as follows: Min *N* = (10/K + 1) × K and Max *N* = (20/K + 1) × K. The original sample size is *K* = 3, the minimum sample size *N* is 13, and the maximum sample size *N* is 23. After considering practical adjustments due to an odd number of groups (3), the actual minimum sample size for the experiment was 15 animals, and the maximum was 24 animals.

In this study, C57BL/6 mice weighing 20–24 g and aged 8–10 weeks, were used. The mice were housed in a controlled environment maintained at a constant temperature of 22 °C with a 12-h light–dark cycle, ensuring *ad libitum* access to food and water. All the experimental procedures adhered strictly to the guidelines of the Experimental Animal Ethics Committee of Fujian Provincial Hospital. The mice were randomly divided into three groups.

### 2.2 Randomization and blinding

Mice were randomly assigned to three groups—right-sided ultrasound-guided SGB (SGB-R), left-sided ultrasound-guided SGB (SGB-L), and control (SGB-Con)—using a computer-generated random sequence in Excel. Each group consisted of eight animals. An independent research assistant prepared the corresponding treatment solutions according to the randomization schedule, and a dedicated technician performed the SGB procedures. All outcome assessments, including heart rate, body temperature, and carotid blood flow velocity and vessel diameter measurements, were conducted by an investigator blinded to the group allocation.

### 2.3 Ultrasound-guided SGB

#### 2.3.1 Selection of ropivacaine injection volume

Currently, standardized dosing guidelines for ultrasound-guided SGB in mice are lacking. Through preliminary dose–response experiments comparing 0.08 mL (12) and 0.10 mL (13, 14) of 0.25% ropivacaine (previously published blind-injection SGB protocols in mice), we found that 0.08 mL reliably achieved complete sympathetic blockade (confirmed by Horner’s syndrome, ipsilateral carotid hemodynamic changes, forelimb temperature elevation, methylene blue tracing, and 3D CT imaging) with no complications, whereas 0.10 mL offered no additional efficacy but increased the risk of brachial plexus blockade and transient respiratory depression. The selected dose of 0.08 mL corresponds to 0.64 mg/kg ropivacaine—far below the reported murine LD_50_ (15) (50–70 mg/kg IV)—providing a wide safety margin. Accordingly, 0.08 mL of 0.25% ropivacaine was adopted as the optimal volume for all subsequent experiments.

#### 2.3.2 Ultrasound-guided SGB protocol

The mice were anesthetized using isoflurane at 5.0% for induction and 1.5%–2.0% for maintenance without endotracheal intubation. The skin surrounding the clavicle, measuring 2 cm by 4 cm, was depilated using a depilatory cream. The mice were placed on their backs on a Vevo 3100 (VisualSonics, FUJIFILM) heating pad operating table with limbs extended and palms secured to the table and connected to heart rate, body temperature, and respiration monitors. After the anesthesia depth stabilized, the procedure commenced. First, using the 5–13-MHz ultrasound probe of the Vevo 3100 system, the pre block inner diameters of the bilateral carotid arteries (averaged from three perpendicular measurements), blood flow velocity (averaged from three 60-degree measurements), limb temperatures, and heart rate were measured.

**FIGURE 1:**
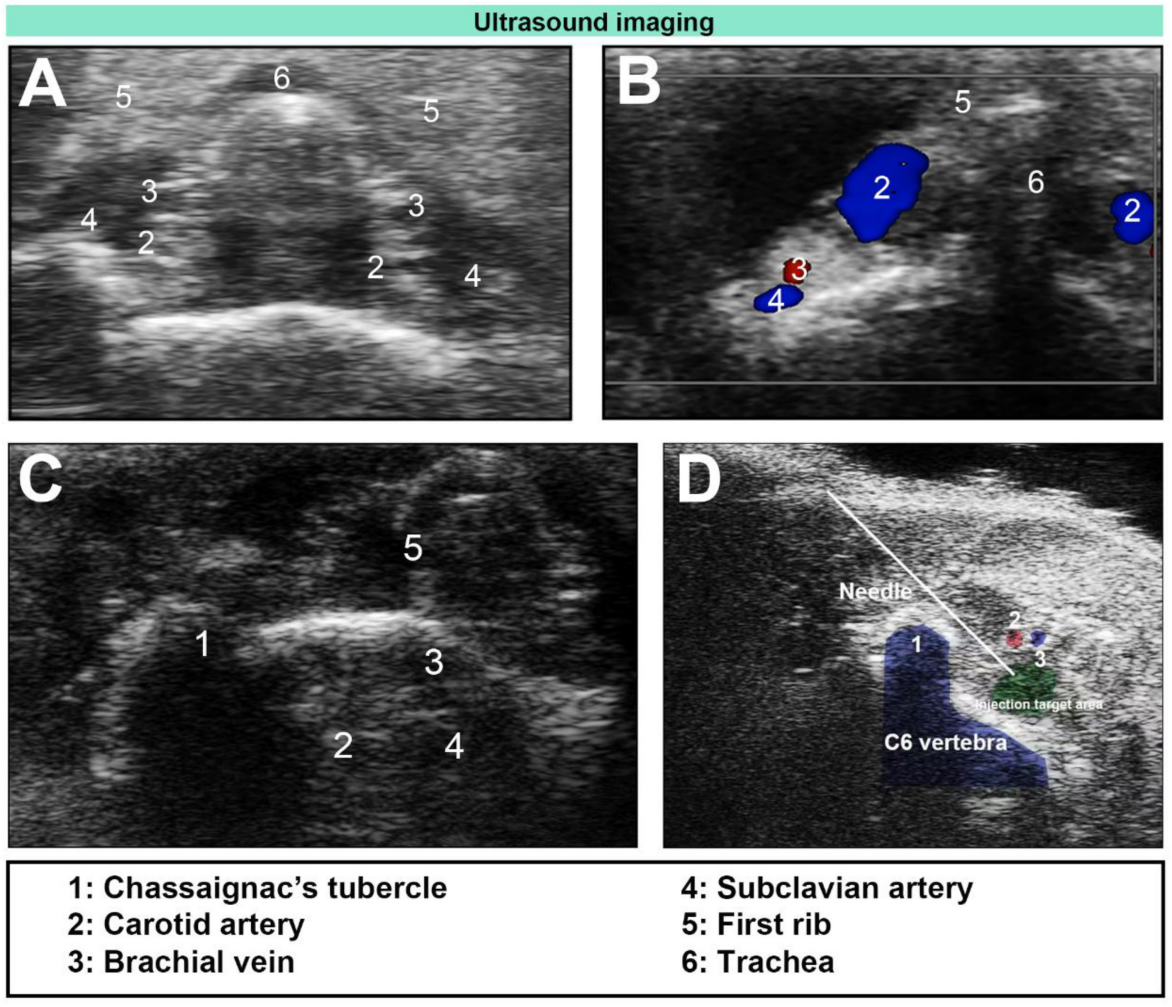
Ultrasound-guided stellate ganglion block (SGB). (A) Ultrasound image of the cervical plane below the level of the first rib in mice. (B) Unilateral Doppler blood flow image. (C) Ultrasound image of Chassaignac’s tubercle and the surrounding vascular anatomy. (D) Ultrasound-guided plane injection (needle).

The ultrasound probe was then positioned at the level of the first rib and moved up and down until the target area-located below the bifurcation of the common carotid arteries and lateral to the trachea-was clearly visualized. This anatomical region corresponds to the vicinity of the anterior tubercle of the C6 transverse process (Chassaignac’s tubercle), a standard landmark for cervical sympathetic blockade (Figures 1A–D). A 1 ml syringe (manufacturer: Henan Dawn Union Bio-Technology Co., Ltd.; registration number: State Medical Device Registration 20153140686) was used, and the needle was advanced carefully to avoid blood vessels and nerves during insertion. Once the needle tip reached the target area, we confirmed that no blood or cerebrospinal fluid could be aspirated before injecting 0.08 ml of 0.25% ropivacaine (manufacturer: AstraZeneca AB; registration number: H20140763). After successful injection, the needle was withdrawn, and pressure was applied to the puncture site. Each mouse was then returned to its cage for observation. If a mouse developed Horner’s syndrome after awakening from anesthesia, SGB was deemed successful (8, 12, 16). Twenty minutes later, the mouse was reanaesthetized and secured to the operating table, and measurements of limb temperature, heart rate, carotid artery diameter, and blood flow velocity were repeated. Following these measurements, the mouse was returned to its cage for continued observation.

### 2.4 Outcomes assessments

#### 2.4.1 Gold standard for success after SGB

**FIGURE 2:**
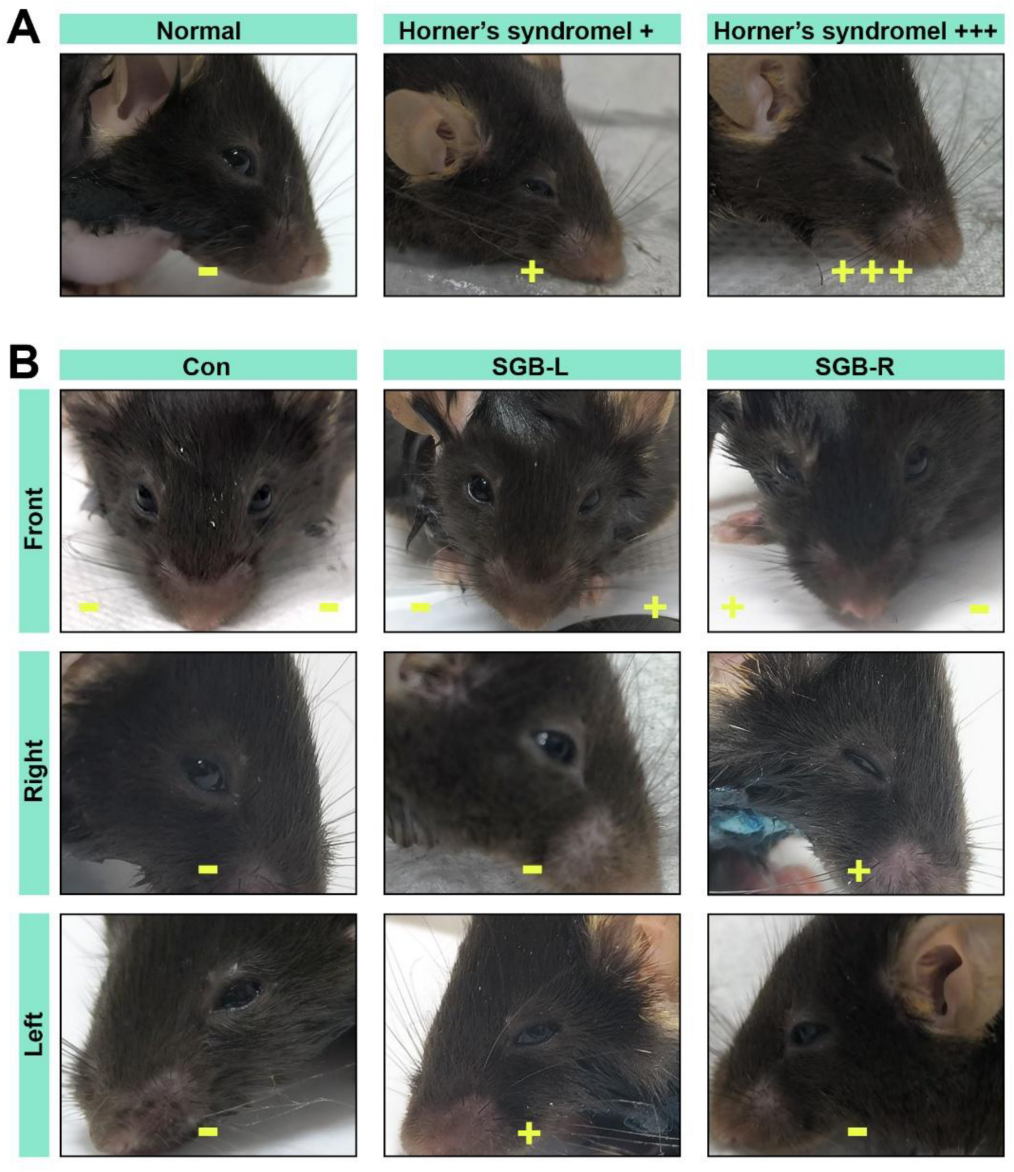
Horner’s syndrome. (A) Diagram illustrating the eyes of C57BL/6 mice in normal and Horner’s syndrome states. (B) Images of Horner’s syndrome from the front, right, and left sides following stellate ganglion block (SGB) under ultrasound guidance in the Con, SGB-L, and SGB-R groups. ^–^No ptosis, ^+^mild ptosis, ^ + ⁣ + +^severe ptosis.

Horner’s syndrome (Figure 2A) can serve as an indicator of the efficacy of stellate ganglion block (SGB) and was assessed as follows, on the basis of the methodology described by Abdi (8): (−): no ptosis; (±): Trace ptosis; (+): mild ptosis; (++): moderate ptosis; and (+++): severe ptosis. The onset of a successful SGB was defined by the appearance of (+) ptosis. The block was considered resolved, marking the duration of effect, when the eyelid returned to its normal position (Figure 2B). All mice exhibiting Horner’s syndrome were included in the statistical analysis.

#### 2.4.2 Carotid artery diameter and blood flow velocity measurement

High-resolution ultrasound imaging was performed using the Vevo^®^ 3100 Imaging System (VisualSonics, FUJIFILM) equipped with a 5–13 MHz linear transducer. Mice were anesthetized with 1.5%–2.0% isoflurane and placed supine on a heated imaging stage, with limb leads attached for continuous ECG monitoring.

The right common carotid artery was imaged in the longitudinal plane at a consistent anatomical location—just proximal to its bifurcation into internal and external branches, approximately 2–3 mm distal to the carotid sinus. To account for cardiac pulsatility, three consecutive measurements of the luminal diameter were made at end diastole (operationally defined as the maximal vessel diameter in the cardiac cycle, synchronized to the R wave on the ECG). Measurements were taken in B mode (2D) by placing calipers on the lumen–lumen interface (intima to intima). The average of the three measurements was used for final analysis.

Pulsed wave Doppler was then applied at the same carotid location to assess blood-flow velocity. The Doppler sample gate was set to 0.3 mm, and the insonation angle was maintained at ≤60°. Three consecutive velocity waveforms were acquired, from which peak systolic velocity (PSV) and time averaged mean velocity (TAMV) were calculated; the mean of the three values for each metric was used. All ultrasound acquisition and measurement procedures were performed by a single operator to minimize inter-operator variability.

### 2.5 Methylene blue tracing for distribution patterns and 3D CT imaging

Following the functional assessment of SGB, an exploratory analysis of SGB distribution patterns will be conducted using methylene blue tracing and 3D CT imaging.

#### 2.5.1 Methylene blue tracing of local anesthetic spread in SGB

Following anesthesia with 5% isoflurane, Methylene blue(A 20 mL stock solution containing 0.25% ropivacaine and 0.1 mg/mL methylene blue was prepared by mixing 5.0 mL of 1% ropivacaine, 0.2 mL of 1% methylene blue, and 14.8 mL of normal saline. A volume of 0.08 mL of this solution was used for each mice injection)was included in the local anesthetic for SGB, In mice exhibiting a successful block, euthanasia was performed by injecting 2 mL of air into the vein, Then, dissection was performed and the spread was quantified. To eliminate systematic bias, the investigators remained blinded to the background during both the planning and assessment of the interventions, and the effectiveness of the blinding was evaluated.

#### 2.5.2 Ultrasound-guided contrast injection and CT 3D imaging

##### 2.5.2.1 Anesthesia induction and maintenance

Mice were initially anesthetized with 5% isoflurane and then maintained on a continuous flow of 1.5%–2.0% isoflurane for the duration of the surgical and imaging procedures. Respiratory rate and pedal reflex were monitored to ensure a stable, adequate depth of anesthesia.

##### 2.5.2.2 Ultrasound-guided injection

Animals were positioned and the target region localized using a 5–13 MHz ultrasound probe on the Vevo 3100 system. Under real-time guidance, the needle was advanced until its tip lay adjacent to the C6 transverse process. After confirming correct placement, the injectate (iohexol diluted at a 1:10 volume ratio, with or without a local anesthetic) was administered.

##### 2.5.2.3 Transition to CT imaging

Immediately after injection, the ultrasound probe and any ancillary apparatus were completely removed from the field of view to prevent artifacts or mechanical obstruction. A preclinical CT scanner (United Imaging uCT 503e) was used to perform time-series scans at predetermined intervals: pre-block (baseline), during stellate ganglion block (immediately post-injection), and at 5 and 12 min post-block. The maintained depth of anesthesia avoided motion artifacts and ensured the needle remained stationary throughout the scan series.

### 2.6 Statistical analysis

Statistical analyses were performed using SPSS version 23.0. Normality of the data was first assessed using appropriate normality tests. For variables that followed a normal distribution, between-group comparisons were conducted using one-way analysis of variance (ANOVA), while within-group comparisons (pre- vs. post-intervention) were analyzed using paired-samples *t*-tests. For variables that did not meet the assumption of normality, non-parametric tests were employed for both between-group and within-group comparisons. A two-tailed *p*-value < 0.05 was considered statistically significant.

## 3 Results

### 3.1 Horner’s syndrome occurred in 100% of the mice following SGB

**FIGURE 3:**
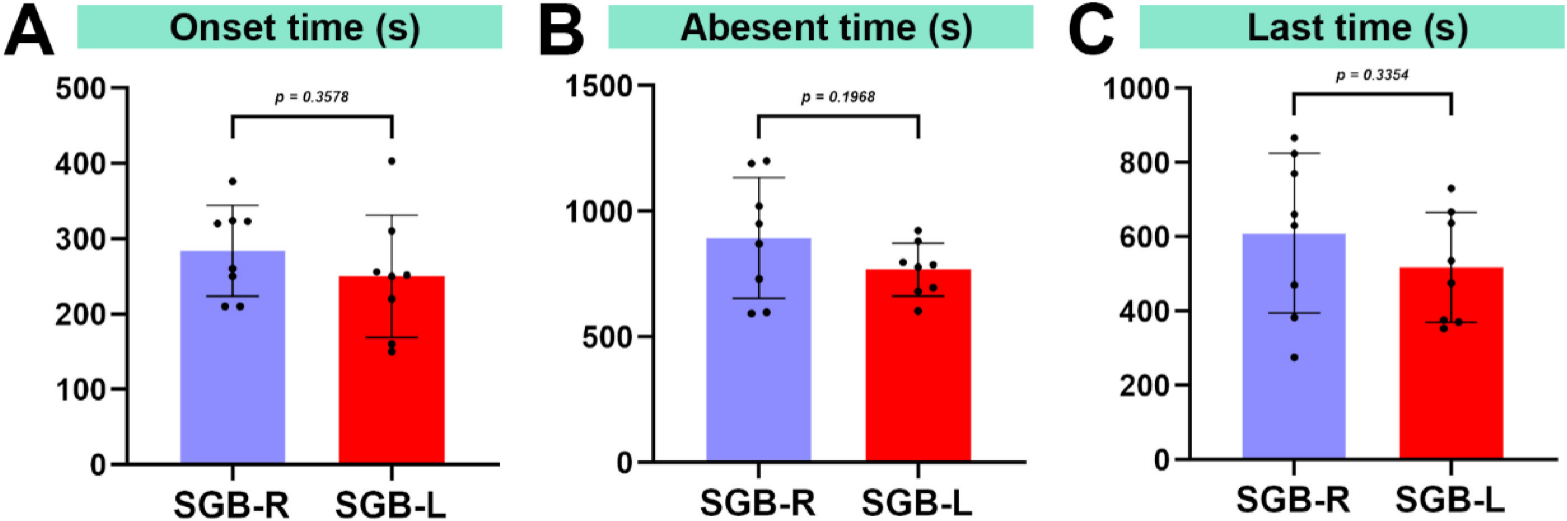
Duration of eyelid ptosis in mice. (A) Time until the occurrence of ptosis following stellate ganglion block (SGB). (B) Time for ptosis resolution after SGB. (C) Duration of ptosis after SGB, *n* = 8.

Upon cessation of isoflurane, the surviving mice reawakened within 160 s. Horner’s syndrome symptoms were first observed in the SGB-right mice at 284.1 ± 60.43 s and in the SGB-left mice at 250.1 ± 81.11 s post-arousal. No signs of Horner’s syndrome were observed in the SGB Con group. The experimental group demonstrated a 100% occurrence rate of Horner’s syndrome, with the incidence rates of mild (+) and severe (++/+++) eyelid ptosis being 6.2% and 93.8%, respectively. No statistically significant differences were detected in either the incidence or duration of eyelid ptosis between the SGB-R and SGB-L groups (Figures 3A–C and Table 1).

**TABLE 1:** The onset, resolution, and duration of Horner’s syndrome, and the number of mice with severe Horner’s syndrome after ultrasound-guided stellate ganglion block (SGB) in the Con, SGB-R, and SGB-L groups.

Group	Onset time (s)	End time (s)	Duration (s)	Horner syndrome+++
**Horner syndrome after ultrasound-guided SGB**				
Con	/	/	/	0
SGB-R	284.1 ± 60.43	893.9 ± 240.8	609.8 ± 214.9	7
SGB-L	250.1 ± 81.11	767.9 ± 105.7	517.8 ± 147.9	8

### 3.2 Validation of SGB argeting and injection accuracy

**FIGURE 4:**
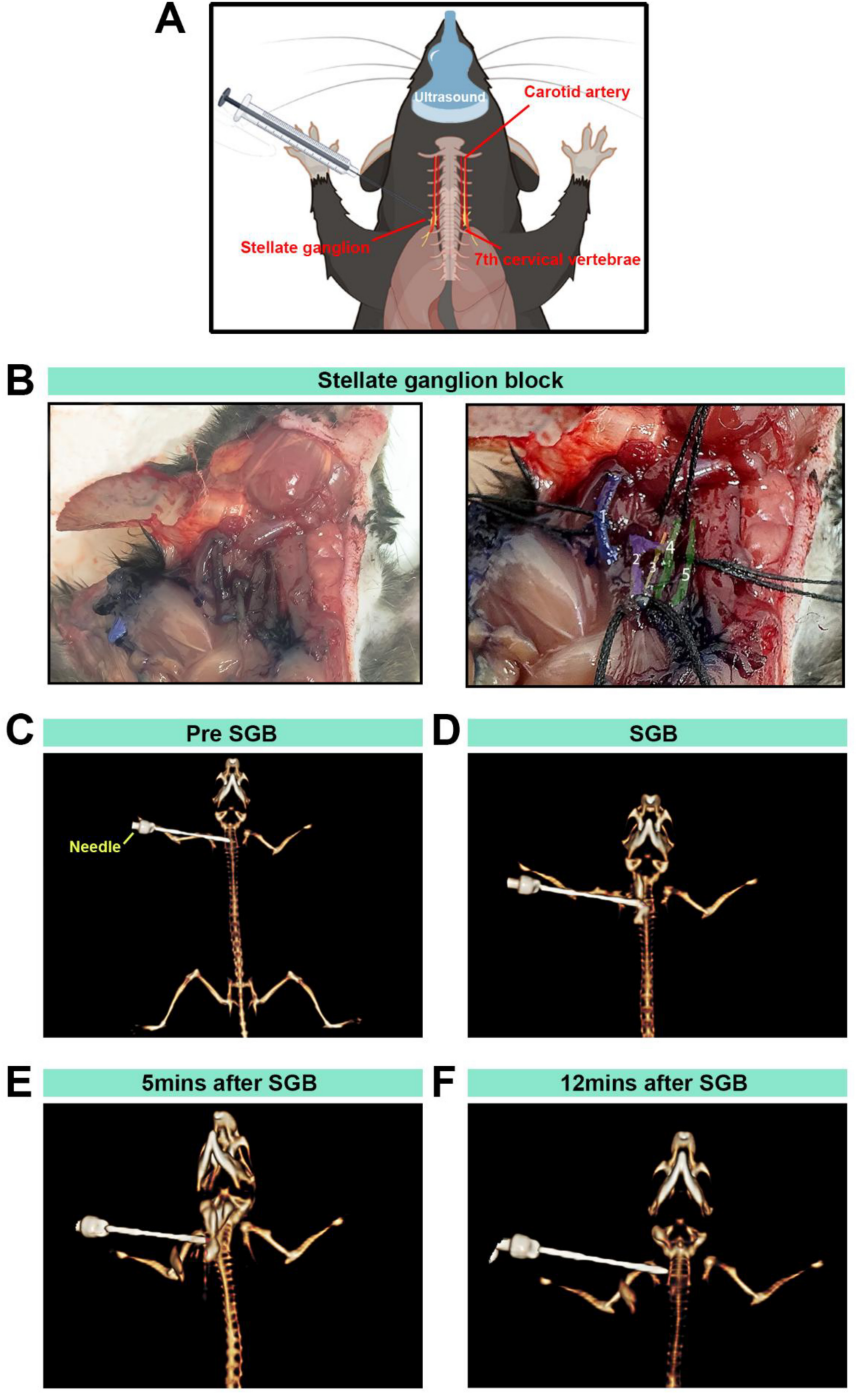
The stellate ganglion block (SGB) model established in mice and anatomical and CT 3D reconstructions. (A,B) Diagram of a stellate ganglion block procedure under ultrasound guidance in C57BL/6 mice, anatomical localization diagram and anatomical localization diagram with pseudocolour labeling: (1) right subclavian vein; (2) right common carotid artery and carotid bifurcation; (3) sympathetic nerve; (4) right brachial vein; and (5) right sternal thyroid muscle. (C) Observation of the positional relationship between the needle tip and the mouse during SGB under 3D reconstruction. (D) CT imaging with three-dimensional reconstruction to observe the distribution of local anesthetic and contrast agents. (E,F) Distribution of the local anesthetic containing contrast agent observed via CT imaging with 3D reconstruction at 5 and 12 min.

The vagal sympathetic chain and carotid artery bifurcation were exposed, revealing the SG below the carotid artery bifurcation, lateral to the trachea (Figure 4A), blue staining was consistently confined to the region surrounding the sympathetic chain adjacent to the C6 transverse process. The spread was quantified, revealing a stained area with a diameter of approximately 1.2 ± 0.3 mm (*n* = 3), with no observable spread to the contralateral side or distal regions. Methylene blue staining verified the accuracy of the procedure (Figure 4B). To further confirm the precision of the injection, a contrast agent was added to the local anesthetic, and the distribution was validated using 3D CT imaging (Figures 4C, D), the contrast agent reached its peak distribution at 5 min post-injection, while it was no longer detectable by 12 min (Figures 4E, F).

### 3.3 Heart rate changes following SGB

**FIGURE 5:**
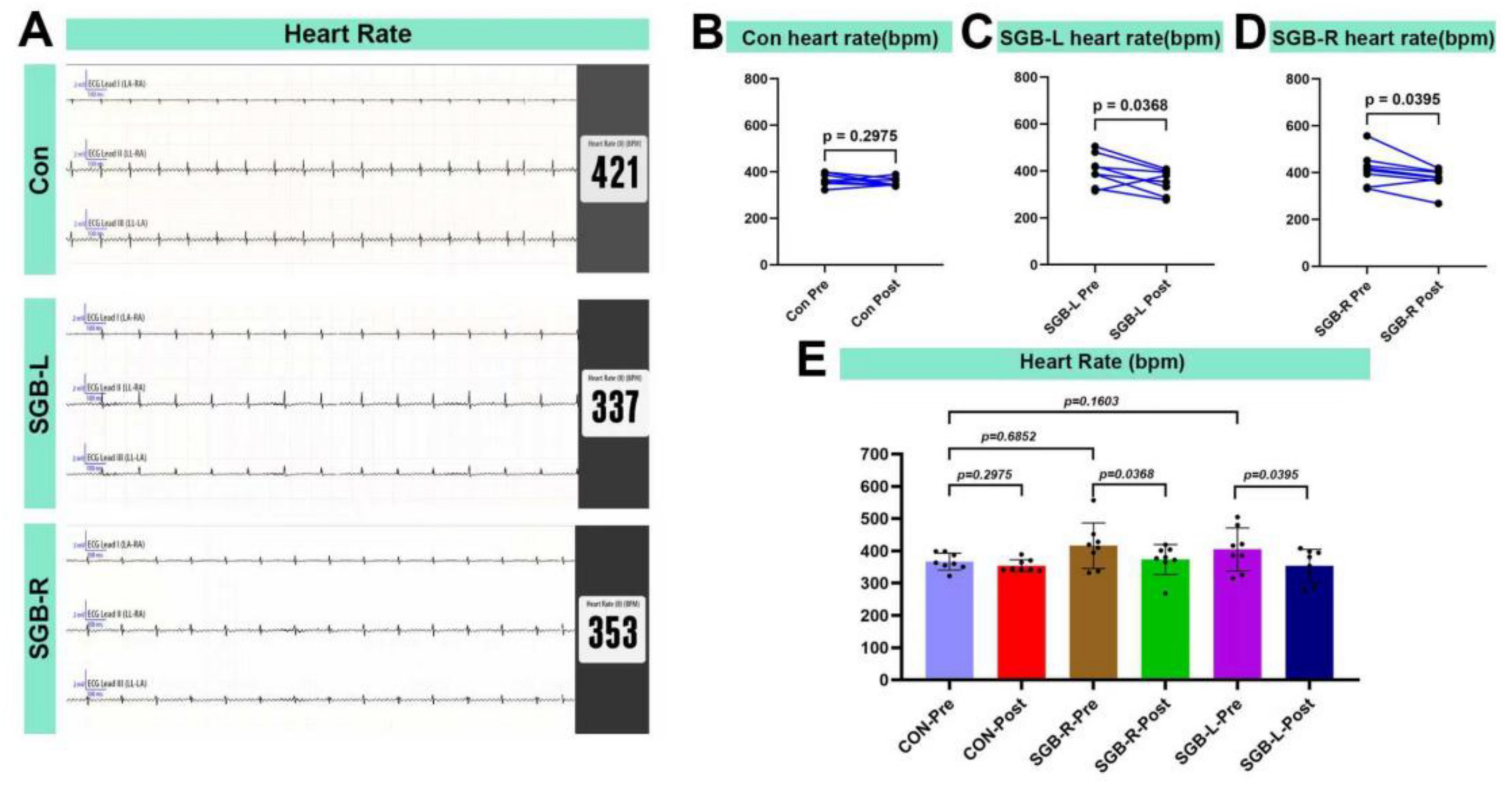
Heart rate reduction after stellate ganglion block (SGB) implementation in a mouse model. (A) Heart rate schematic for three groups of mice. (B–D) Changes in heart rate before and after block in mice in the Con, SGB-R, and SGB-L groups, *P* < 0.05, *n* = 8. (E) Changes in heart rate before and after block in the Con, SGB-R, and SGB-L groups, *P* < 0.05, *n* = 8.

At 20 min after SGB intervention, the heart rates of all surviving mice were evaluated (Figure 5A). There were no significant differences in heart rate before SGB among the Con, SGB-L, and SGB-R groups. Heart rates in the SGB-R-post and SGB-L-post groups were lower compared to SGB-R-pre and SGB-L-pre, and the differences were statistically significant (*p* < 0.05), while no change was observed in the Con group (Figures 5B–E and Table 2).

**TABLE 2:** There were no significant differences in heart rate before stellate ganglion block (SGB) among the Con, SGB-L and SGB-R groups.

Variable	Con	SGB-L	SGB-R	*F*-value	*P*-value
**One-way ANOVA of the three groups before SGB**					
Heart rates before SGB	366.6 ± 26.3	404.3 ± 66.8	416.1 ± 70.9	1.576	0.230

### 3.4 Temperature changes following SGB

**FIGURE 6:**
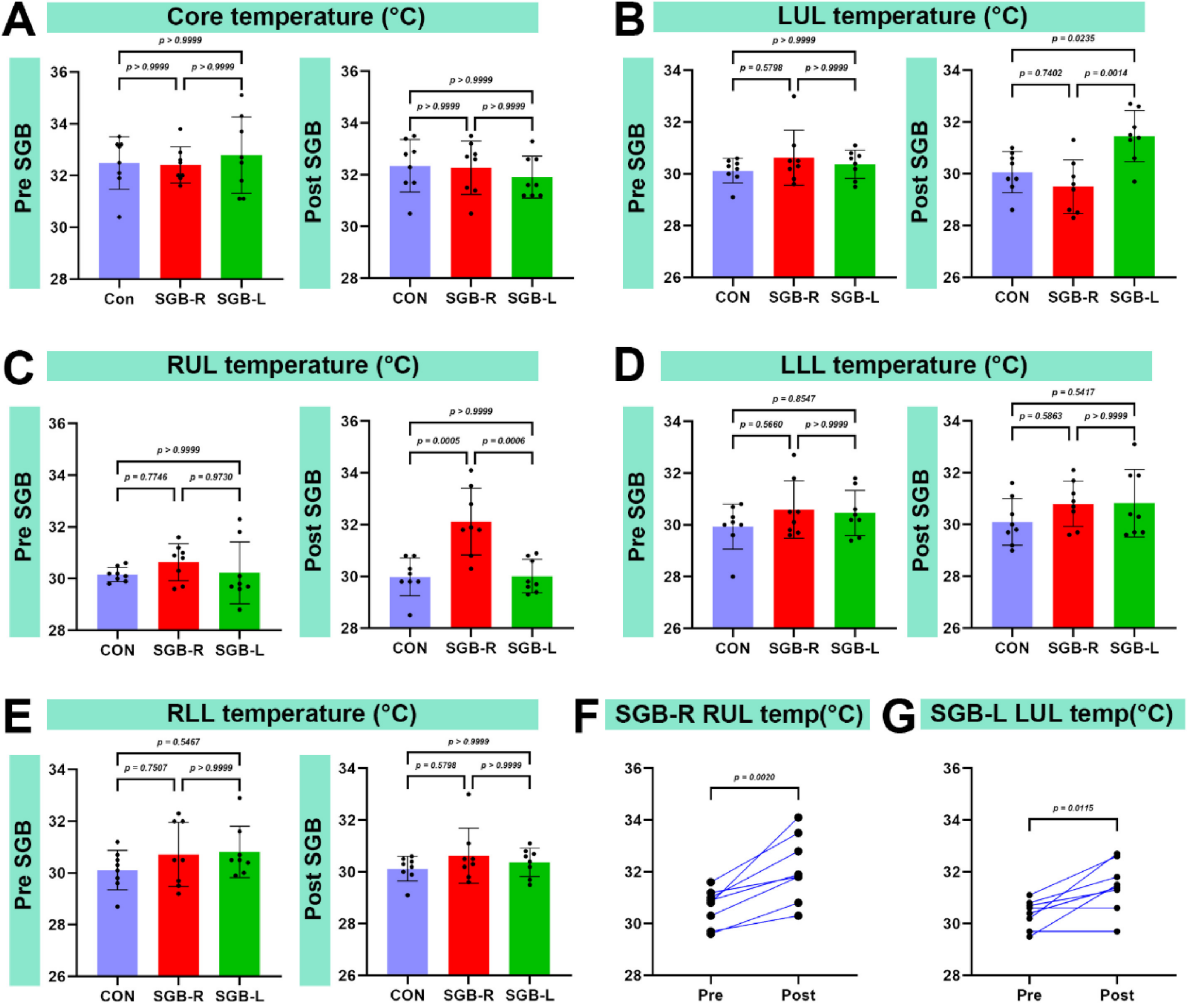
Temperature of the upper limb on the blocked side. (A) Changes in the core temperature of C57BL/6 mice before and after ultrasound-guided stellate ganglion block (SGB). (B) Changes in the left upper limb temperature of C57BL/6 mice before and after ultrasound-guided SGB. (C) Changes in the right upper limb temperature of C57BL/6 mice before and after ultrasound-guided SGB. (D) Changes in the left lower limb temperature of C57BL/6 mice before and after ultrasound-guided SGB. (E) Changes in the right lower limb temperature of C57BL/6 mice before and after ultrasound-guided SGB. (F,G) Temperature changes in the right and left upper limbs of mice before and after SGB, *P* < 0.05, *n* = 8.

There was no significant difference in core temperature among the Con, SGB-R, SGB-L groups (Figure 6A). The temperature of the blocked upper limb was greater in the SGB-R and SGB-L groups than in the Con group (*p* < 0.05; Figures 6B, C). However, the lower limb temperatures did not differ between the SGB-R and SGB-L groups (Figures 6D, E). Moreover, The temperature of the ipsilateral upper limb (right limb in the SGB-R group, left limb in the SGB-L group) was higher post-SGB than pre-SGB in both the SGB-R and SGB-L groups, and the differences were statistically significant. The temperature of the right upper limb after right-sided SGB was higher than that before the block, and the temperature of the left upper limb after left-sided SGB was higher than that before the block, with a statistically significant difference. (Figures 6F, G).

### 3.5 Changes in the internal diameter of the carotid artery following SGB

**FIGURE 7:**
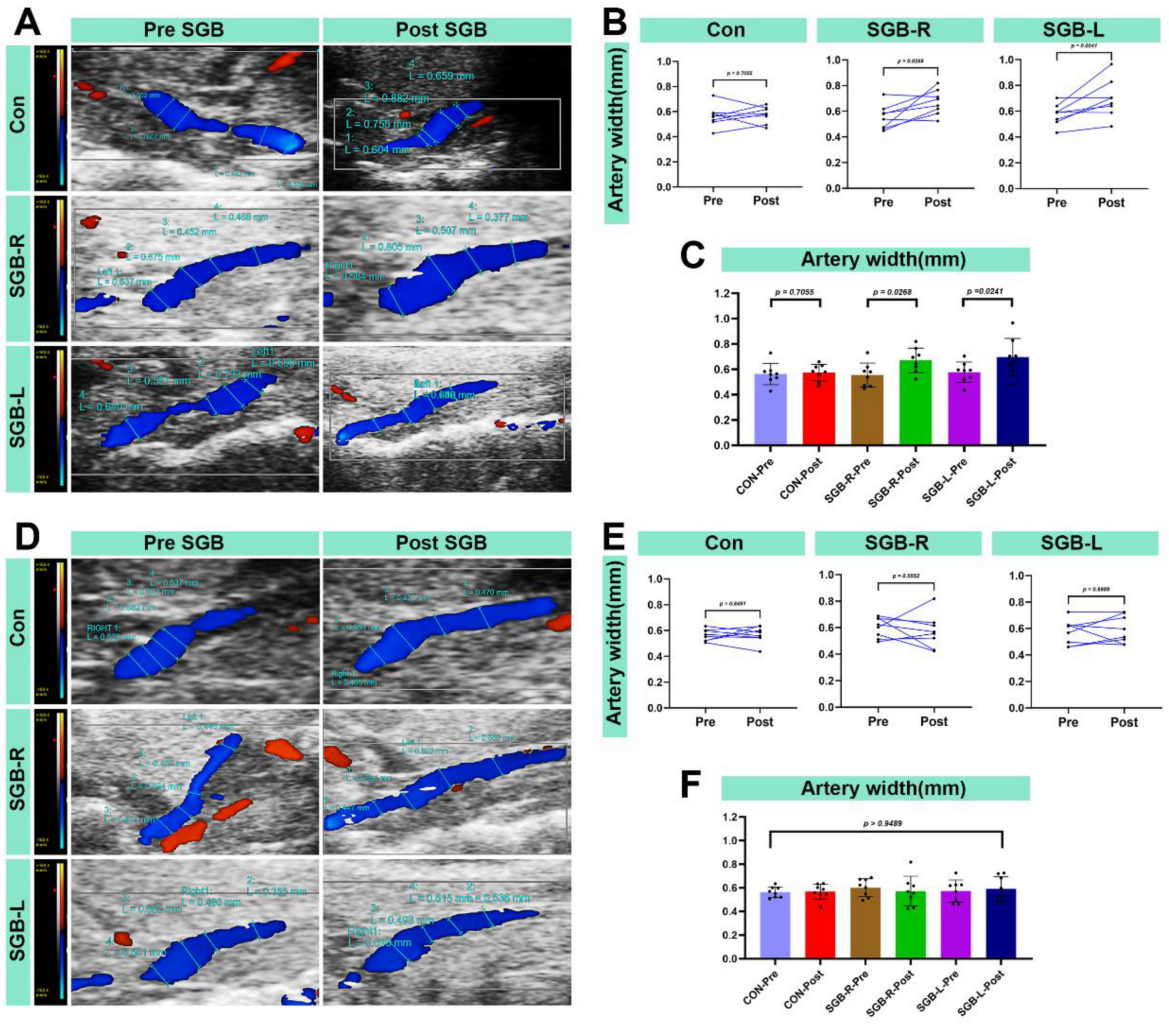
Increased blood flow diameter in the carotid artery after stellate ganglion block (SGB) implementation in a mouse model. (A) Diagram of changes in the ipsilateral carotid artery diameter of C57BL/6 mice before and after ultrasound-guided SGB (with unilateral measurements in the Con group). (B,C) Differences in the carotid artery diameter in the ipsilateral carotid artery of C57BL/6 mice before and after ultrasound-guided SGB (with unilateral measurements in the Con group), *P* < 0.05, *n* = 8. (D) Diagram of the carotid artery diameter in the contralateral carotid artery of C57BL/6 mice before and after ultrasound-guided SGB (with unilateral measurements in the Con group). (E,F) Differences in the carotid artery diameter in the contralateral carotid artery of C57BL/6 mice before and after ultrasound-guided SGB (with unilateral measurements in the Con group), *P* < 0.05, *n* = 8.

All surviving mice underwent carotid artery monitoring 20 min after SGB. The ipsilateral carotid artery diameter was larger after SGB than before in both the SGB-R and SGB-L groups. Furthermore, this diameter was greater than in the Con group, and the differences were statistically significant (*p* < 0.05; Figures 7A–C). No significant changes were observed in the contralateral carotid artery diameter in all groups (Figures 7D–F).

### 3.6 Carotid blood flow velocity following SGB

**FIGURE 8:**
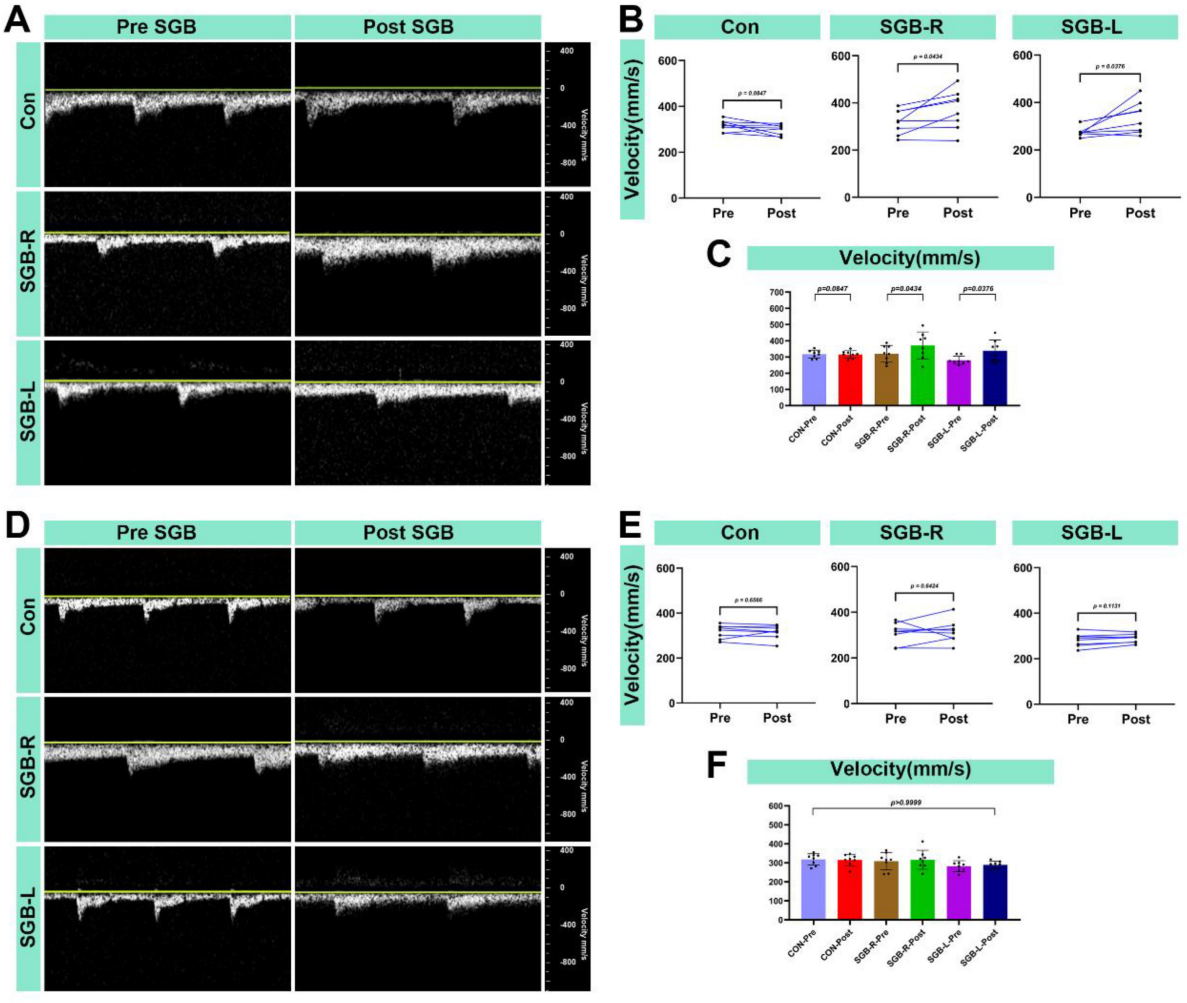
Increased blood flow velocity in the carotid artery after stellate ganglion block (SGB) implementation in a mouse model. (A) Diagram of changes in the ipsilateral carotid artery blood flow velocity of C57BL/6 mice before and after ultrasound-guided SGB (with unilateral measurements in the Con group); (B,C) Differences in the changes in the blood flow velocity in the ipsilateral carotid artery of C57BL/6 mice before and after ultrasound-guided SGB (with unilateral measurements in the Con group), *P* < 0.05, *n* = 8; (D) Diagram of the carotid blood flow velocity in the contralateral carotid artery of C57BL/6 mice before and after ultrasound-guided SGB (with unilateral measurements in the Con group); (E,F) Differences in the changes in the blood flow velocity in the contralateral carotid artery of C57BL/6 mice before and after ultrasound-guided SGB (with unilateral measurements in the Con group), *P* < 0.05, *n* = 8.

All surviving mice underwent blood flow velocity monitoring 20 min after the SGB intervention. The ipsilateral carotid artery blood flow velocity was higher after SGB than before in both the SGB-R and SGB-L groups. There was no significant change in blood flow velocity in the Con-post. When compared to the Con group, both the SGB-R and SGB-L groups showed an increase in blood flow velocity, and the differences were statistically significant (*p* < 0.05; Figures 8A–C). No significant changes were detected in the contralateral carotid artery in any of the groups (Figures 8D–F).

### 3.7 Complications

No obvious postoperative complications (brachial plexus block, brachial plexus injury, hematoma, respiratory distress or death) occurred in any of the three groups of mice.

## 4 Discussion

Compared with rats, mice have more pronounced advantages as model animals, such as a greater variety of genetically modified strains, including more types of knockout and knock-in strains, and a larger number of strains available for the establishment of various experimental animal models; in addition, mouse models are frequently utilized in immunological research and tumor modeling (17–19). While ultrasound-guided SGB has been used in rats (7), this study is the first to describe its use in mice. Similar to that in rats (7), the SG in mice consists of the inferior cervical ganglion and the T1 to T3 ganglia (7, 20, 21), which are located from the C7 to T1 vertebrae (13, 21). In mice, the longissimus cervicis muscles are situated adjacent to both sides of the vertebral column. In this study, the anterior tubercle of the C6 transverse process was utilized as a landmark for needle positioning. The needle was inserted along the lateral border of the C6 vertebral body, near the stellate ganglion. During injection, the bevel of the needle was directed laterally to administer the local anesthetic slowly, ensuring its localization around the stellate ganglion. Ropivacaine, a long-acting amide-type local anesthetic, is selected for its prolonged effect and lower toxicity. On the basis of previous studies (13, 22) and preliminary experimental results, 0.25% ropivacaine at a volume of 0.08 ml was utilized to perform SGB in this study, introducing a new method for SGB in mice and verifying its accuracy. Using ultrasound guidance, significant increases in carotid artery diameter and blood flow velocity were observed. The precise spread of the local anesthetic and the position of the needle tip were confirmed through 3D CT imaging and anatomical verification. These findings establish the effectiveness and safety of this technique.

The SG modulates sympathetic activity, leading to a decreased heart rate. In our study, There was no statistically significant difference in heart rate among the Con-pre, SGB-R pre, and SGB-L pre groups, indicating that the baseline heart rates were comparable across groups. the postintervention heart rates in the SGB-R and SGB-L groups decreased significantly compared with their preintervention values. Our study findings are consistent with the general trends observed by Duan et al., although there are notable differences in statistical outcomes. In Duan et al.’s study, while a decreasing trend in heart rate was observed in the SGB-L group, this change did not reach statistical significance. In contrast, our study demonstrated a statistically significant reduction in heart rate following SGB-L intervention. This finding aligns with clinical research evidence, which has confirmed that SGB-L effectively reduces heart rate (23, 24). Some studies indicate that the nerve fibers of the cervical ganglia may traverse within the same connective tissue sheath as the vagus nerve (25–27). Consequently, while the vagus nerve may also be subject to blockade during SGB administration, sympathetic nerve blockade is likely more pronounced, potentially causing a greater sympathetic effect than a vagal effect (28). Additionally, the SG contains both sympathetic and vagal nerve fibers, contributing to the complex crosstalk between these systems (28).

In our study, mice in both the SGB-R and SGB-L groups developed Horner’s syndrome following ultrasound-guided SGB. Although the duration of Horner’s syndrome lasts only about 10 min, the pharmacodynamic effect of ropivacaine on sympathetic nerve blockade can last for several hours, The brief functional effect we observed can be attributed to the extremely high metabolic rate and rapid drug clearance in mice. The duration of Horner’s syndrome in our study was shorter than that reported in previous research protocols (13), which may be attributed to the use of ultrasound-guided SGB in our approach, as opposed to blind injection techniques used in earlier studies. Additionally, differences in injection volume (0.1 mL vs. 0.08 mL) may also contribute to this observation. Compared with the Con group, the experimental groups presented increased carotid artery diameters and blood flow velocities 20 min post block. These changes can be attributed to the vasodilation that occurs when the sympathetic nerve supply to the vessel is interrupted. Specifically, we observed the following: (1) Increased blood flow velocity: This is likely due to reduced sympathetic tone, resulting in decreased vascular resistance and subsequently increased blood flow velocity in the carotid artery. (2) Increased internal diameter: This is a result of vasodilation leading to the expansion of the carotid artery’s internal diameter. These findings support the effectiveness and accuracy of the ultrasound-guided technique for SGB. While vascular morphometric analysis is widely employed across disciplines, standardized measurement protocols are essential to ensure reliable hemodynamic assessment. In this study, carotid artery diameters were consistently measured at end-diastole—when the lumen is maximal—to minimize variability caused by cardiac pulsatility (29–31). Although such precise, phase-locked measurements have not been extensively reported in prior SGB studies, they provide critical insights into sympathetic regulation of arterial tone. However, clinical studies in humans have demonstrated that SGB has analogous effects. A study examined 19 healthy female volunteers before and after SGB using a 1.5-T MRI. The results revealed that signal intensity changes were primarily noted in the ipsilateral extracranial vessels, specifically the external carotid artery and its downstream branches, including the occipital artery and the superficial temporal artery. Conversely, the intracranial arteries’ intensities remained unchanged, except for those of the ipsilateral ophthalmic artery, which demonstrated a significant increase. Additionally, post-SGB, only the diameter of the ipsilateral external carotid artery was significantly increased (32). This phenomenon may result from the modulation of peripheral arteries and arterioles by sympathetic nerves. Additionally, compared with the Con group, the SGB-R and SGB-L groups presented an increase in the temperature of the ipsilateral upper limb, likely a consequence of vasodilation induced by sympathetic blockade. This temperature change validates the accuracy and effectiveness of the ultrasound-guided SGB.

Pneumothorax, nerve block or damage, and hemorrhage are severe complications of SGB that may significantly influence experimental results and research findings (33, 34). Some studies have indicated that nerve block-related harm is predominantly attributed to mechanical trauma, applied pressure, and neurotoxicity (35–37). The extent of nerve injury during nerve block procedures is influenced by both the puncture angle and needle size. Visual guidance offers clear visualization of the needle’s position, insertion angle, and drug diffusion, thereby minimizing mechanical injury by ensuring precise needle placement (38). Ultrasound technology provides clear visualization of anatomical structures—including muscles, nerve and surrounding vessels—thereby improving needle-navigation accuracy and increasing the success rate of nerve blocks (39, 40). In our study, no complications or deaths occurred among the 24 mice undergoing ultrasound-guided SGB, whereas a previous blind SGB study reported an 18.4% overall complication rate, including brachial plexus block (12.3%), vascular injury (4.6%), and mortality (1.5%) (13). These results indicate that ultrasound-guided SGB has a markedly superior safety profile in mice. This improvement is largely attributable to the unique advantages of real-time ultrasound: (1) precise visualization of the stellate ganglion and adjacent structures such as the common carotid artery, internal jugular vein, and pleural dome, which reduces off-target injection and puncture risks; (2) dynamic needle tracking that ensures accurate periganglionic drug delivery and minimizes operator variability; and (3) Doppler imaging that further enhances vascular identification and anatomical localization during the procedure.

It is crucial to acknowledge the limitations of our study. First, Although ultrasound allows clear identification of the key anatomical landmarks surrounding the SG, the SG itself cannot be directly visualized or accurately localized. Second, ultrasound-guided SGB surgeries require high-frequency ultrasound equipment, which reduces their portability compared with blind detection methods. Third, a change in facial temperature is a classic sign of stellate ganglion block; however, because mice have small faces that are covered in fur, accurate measurement of facial temperature is challenging. Therefore, the temperature at the distal extremities was used as a substitute. Fourth, The post-SGB time points (5 and 12 min) were chosen based on preliminary data showing peak contrast distribution at 5 min and resolution by 12 min. However, dynamic, real-time tracking of contrast diffusion was not possible due to CT setup limitations. Although cross-sectional images could enable detailed analysis, they were severely compromised by needle-induced metal artifacts in the small C57BL/6 mice, exacerbated by the spatial resolution limits of our preclinical scanner. Consequently, robust cross-sectional analysis was unfeasible, and we relied on 3D reconstructions for gross localization.

## 5 Conclusion

This study provides a methodological blueprint for performing ultrasound-guided stellate ganglion block in C57BL/6 mice, demonstrating its effectiveness and safety. The resulting SGB model is highly stable and exhibits minimal complications. Compared to traditional methods, this approach is better suited for research involving SGB.

## Data Availability

The original contributions presented in this study are included in this article/supplementary material, further inquiries can be directed to the corresponding authors.
